# Physico-Chemical Properties and Phase Behavior of the Ionic Liquid-β-Cyclodextrin Complexes

**DOI:** 10.3390/ijms140816638

**Published:** 2013-08-13

**Authors:** Marek Rogalski, Ali Modaressi, Pierre Magri, Fabrice Mutelet, Aleksandra Grydziuszko, Michał Wlazło, Urszula Domańska

**Affiliations:** 1 LCP-A2MC, EA 4164, Université de Lorraine, 1, bd Arago-57078 METZ, Cedex 3, France; E-Mails: rogalski@univ-metz.fr (M.R.); modaress@univ-metz.fr (A.M.); magri@univ-metz.fr (P.M.); 2 Institut National Polytechnique De Lorraine, Nancy Université, 2 av.de la Forêt de Haye BP3, Vandceuvre-lès-Nancy Cedex 54501, France; E-Mail: fabrice.mutelet@univ-lorraine.fr; 3 Department of Physical Chemistry, Faculty of Chemistry, Warsaw University of Technology, Noakowskiego 3, Warsaw 00-664, Poland; E-Mails: akropiewnicka@gmail.com (A.G.); mwlazlo@ch.pw.edu.pl (M.W.)

**Keywords:** β-cyclodextrin-ionic liquid systems, experimental solid-liquid equilibrium, activity coefficients at infinite dilution, intermolecular interactions

## Abstract

The solubility of β-cyclodextrin (β-CD) in ionic liquids (ILs) and the activity coefficients at infinite dilution ( $\gamma_{13}^{\infty }$) of more than 20 solutes (alkanes, aromatic hydrocarbons, alcohols) were measured in four chosen ionic liquids, their mixtures with β-CD, and in the β-CD at high temperatures from 338 to 398 K using the inverse gas chromatography. The intermolecular interactions, inclusion complexes and the possible increasing of the solubility of β-CD in water using the IL are presented. The solubility of β-CD in ten chosen hydrophobic ILs at the temperature *T* = 423 K was detected. The solid-liquid phase diagrams (SLE) of {IL (1) + β-CD (2)} binary systems at the high mole fraction of the IL were measured for three systems (1-ethyl-3-methylimidazolium chloride, [EMIM][Cl], 1-ethyl-3-methylimidazolium bromide, [EMIM][Br]; and for 1-butyl-3-methylimidazolium chloride, [BMIM][Cl]). The eutectic points were determined at the high IL concentration for all binary systems. The intermolecular interaction and the possibility of inclusion complexes of the IL and/or solvents with β-CD were discussed. The infrared spectroscopy, IR was used for the description of the intermolecular interactions in the (β-CD + IL) systems. It was shown via the activity coefficients at infinite dilution results that the inclusion complexes are dependent on the temperature. The addition of β-CD to the IL does not improve the selectivity of the separation of the aliphatics from aromatics.

## 1. Introduction

Numerous compounds–supramolecular guests of different shape and polarity, ions, and even radicals are known to form stable inclusion complexes with β-cyclodextrin (β-CD), a well-known host molecules [1–8]. Its relatively non-polar cavity is capable of forming inclusion complexes with a variety of molecules. Thus, this ability can be exploited in many areas such as cosmetics, foods, pharmaceuticals, biotechnology, and physiological systems [9,10]. The β-CD is mainly utilized to improve aqueous solubility and bioavailability. For example, recently, the interaction of β-CD with one component monolayer of cholesterol mixtures has been investigated in the aqueous sub-phase [11]. This cyclic molecule forms a cone-shaped cavity with hydrophilic outer and a relatively hydrophobic inner surfaces [12]. Usually, the less polar part of the guest molecule is accommodated by the cavity, and the polar part is exposed to the aqueous environment. The aqueous solubility of β-CD is very small and is about 18.5 g·dm^−3^ (0.016 mol·dm^−3^) at room temperature. It was shown that β-CD exists in the aqueous solution as aggregates [13]. Therefore, the inclusion complexes are formed with free cyclodextrin molecules. Usually, the aggregates and colloidal particles are present in the aqueous solutions.

In recent years, great progress has been made in chemistry and physico-chemical properties of ionic liquids (ILs) [14–20]. Imidazolium-based ILs with a different anion are treated as a novel solvents with interesting properties, which can be used as an entrainers in the separation processes for the several mixtures [15,16,19,20]. The measurements of activity coefficients at infinite dilution present the first information about the interaction between the IL and the solvent. The selectivity parameter, calculated from the $\gamma_{13}^{\infty }$ data gives the information that IL is attractive for the chosen separation process. The important petroleum problem is the extraction of aromatic hydrocarbons from aliphatic hydrocarbons, or of thiophene from aliphatic hydrocarbons with the high selectivity. The results are usually compared to sulpholane or *N*-methylpyrrolidinone (NMP), which are used in the industry [15,16,19,20]. The interaction in such a liquid mixture is influenced by dispersion forces, dipole-dipole interaction, hydrogen bonding, charge transfer, and many other factors. The application of ILs is of practical importance in extraction, solar batteries, lubrications, adhesion, catalysis, and many other possible technological uses [21]. The physico-chemical properties of ILs depend on the polymorphic forms and the cation/anion combination [15,16].

ILs may be described in solid, or liquid states as hydrogen-bonded polymeric supramolecules. The introduction of other molecules and macromolecules occurs with a disruption of the hydrogen bond network and in some cases can generate nanostructures with polar and non-polar regions where inclusion-type compounds can be formed [22].

In the past, different physico-chemical methods were used to proof the association binding constants between CD and ILs [23–27]. Near infrared spectrometry was used to determined complexes of phenol with CD in 1-butyl-3-methylimidazolium chloride, [BMIM][Cl], [23]. The solubility and conductivity measurements were used in aqueous solutions of β-CD and 1-butyl-3-methylimidazolium hexafluorophosphate, [BMIM][PF_6_] [24]. The surface tension measurements were used to observe inclusion complexes of β-CD and the imidazolium surfactants [25]. The solubilities and ^1^H NMR and IR spectroscopic measurements were used to study the enhanced solubility of β-CD in dicyanamide-based ILs [26]. Recently, the *α*-, β-, and *γ*-cyclodextrins interaction with different cations, for example, the 1-octyl-3-methylimidazolium cation, [OMIM]^+^, and anions as tetracyanoborate [TCB]^−^, {bis(trifluoromethyl)sulfonyl}imide [NTf_2_]^−^, or tris(pentafluoroethyl)trifluorophosphate, [FAP]^−^ were described using NMR spectra and isothermal microcalorimetry titration [27]. Simple one-point information about the solubility of CDs in imidazolium-based ILs is also recently presented in a review of the solubility of carbohydrates in ILs [28].

This paper is a continuation of our earlier investigation into the inclusion complexes with β-CD [29]. The present work provides new insights into the effect of β-CD on the interaction with the IL at the specific binding stage, which contributes to understanding the effect of cyclodextrin on the properties of the solution and functionality of the {β-CD + IL} complexes. Using 1-ethyl-3-methylimidazolium chloride, [EMIM][Cl], 1-ethyl-3-methylimidazolium bromide, [EMIM][Br], [BMIM][Cl], 1-butyl-1-methylpyrrolidinium chloride, [BMPYR][Cl], and many others ILs, the one-temperature solubility was determined. The SLE for chosen systems, and the activity coefficients at infinite dilution measurements, were presented for many polar and non-polar solvents. The characteristic investigated here includes the effect of the type of the IL (different anions and cations), the intermolecular interaction of different solvents with mixture of the IL with β-CD and with pure β-CD. The inclusion complexes of β-CD with different ILs and/or solvents are discussed. The possibility of increasing the solubility of β-CD in water using the IL is proposed. Different mechanism of inclusion complexes with the guest is discussed.

## 2. Results and Discussion

### 2.1. Solubility Measurements

β-CD has many advantages, as was mentioned in the introduction, but is immiscible with most of the solvents, besides with dimethylsulphoxide (DMSO) and dimethylsulphide (DMS). The ILs chosen in this work were expected to have the possibility of forming inclusion complexes with β-CD and to be better solvents than many other because of their differences in polarity and the specific interaction possibilities. In the first step of measurements, the following ILs were tested in the temperature *T* = 423 K: 1-ethyl-3-methylimidazolium chloride, [EMIM][Cl]; 1-ethyl-3-methylimidazolium bromide, [EMIM][Br]; 1-butyl-3-methylimidazolium chloride, [BMIM][Cl]; 1-ethanol-3-methylimidazolium chloride, [EtOHMIM][Cl]; 1-butyl-1-methylpyrrolidinium chloride, [BMPYR][Cl]; 1-butyl-1-methylpyrrolidinium bromide, [BMPYR][Br]; 1-butyl-3-methyimidazolium tetrafluoroborate, [BMIM][BF_4_]; [BMIM][PF_6_]; 1-octyl-3-methylimidazolium bis{(trifluoromethyl)sulfononyl}imide, [OMIM][NTf_2_], and 1-butyl-1-methylpyrrolidinium triflate, [BMIM][CF_3_SO_3_].

It was observed that only four of these ILs reveal homogenic one-phase solution at the measured temperature: [EMIM][Cl], [EMIM][Br], [BMIM][Cl], and [BMPYR][Cl]. One of these ILs shows liquid-liquid binary phase solution ([BMIM][CF_3_SO_3_]) and the other were composed of two phases, liquid and solid.

**Figure 1:**
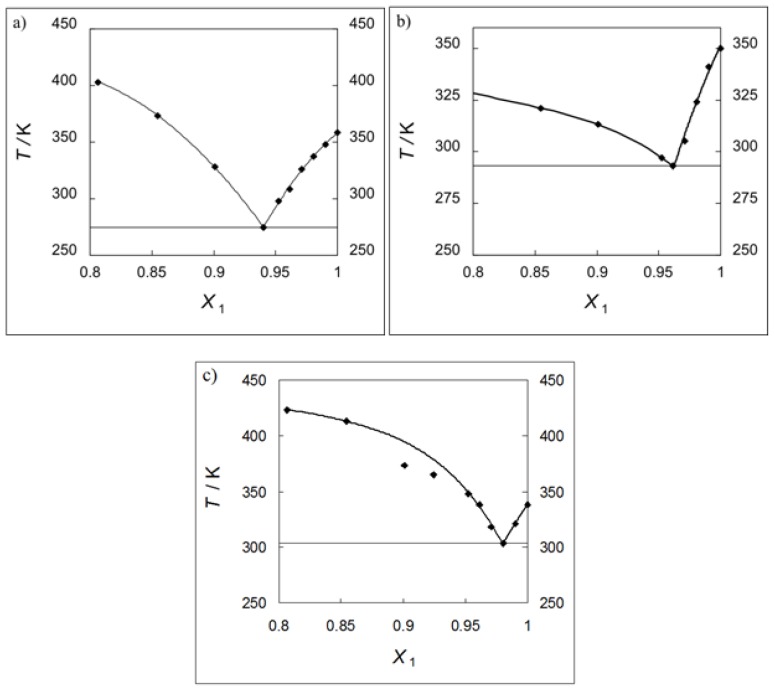
SLE phase diagrams of the binary mixtures: (**a**) {[EMIM][Cl] (1) + β-CD (2)}; (**b**) {[EMIM][Br] (1) + β-CD (2)}; (**c**) {[BMIM][Cl] (1) + β-CD (2)}.

From the results of the one-temperature solubility measurements, the three ILs were chosen for the systematic solid-liquid equilibrium (SLE) measurements at the IL reach phase. The ILs without very high melting temperatures were chosen: [EMIM][Cl], [EMIM][Br], and [BMIM][Cl]. In was shown that the small amount of the β-CD added to the IL decreases the equilibrium temperature of the saturated solution and produces the eutectic point (see Figure 1a–c and Table S1). The eutectic compositions, *x*_1,e_ are between 0.94 and 0.98 mole fraction of the IL and the temperatures of the eutectic points are between 275.0 and 302.5 K, respectively, for the investigated ILs (see Table S2). The solubility of β-CD in [BMIM][Cl] was shown in literature as 21 wt.% at a not defined temperature, which is difficult to compare to our results ([28] and literature cited in). The melting temperatures of the ILs, described in the Table S1 differ slightly from the literatures values. The data from the literature are: 362.15 K [30], 352.15 K [30], and 341.95 K [31], for [EMIM][Cl], [EMIM][Br], and [BMIM][Cl], respectively. These differences are probably due to the synthesis of the IL, purity (water contamination) and very often depend on the producer.

The β-CD cavity may include only the alkane chain of the IL inside cavity. However, the polarity of the ring of the β-CD outside surface is able to associate imidazolium ring. The alkane-chain of the IL may migrate in the cave and the imidazolium cation may be solvated anywhere outside the cavity. Usually, the inclusion complexes of (IL + β-CD) are 1:1 as the result of inclusion force, ordering and/or density in the cave and in the bulk polar region around the cave in the high mole fraction of the IL region. These forces are evidently more significant than van der Waals forces, thus the inclusion compounds have stability. Such complexes were earlier described in water [32].

The interaction pattern of different ILs with β-CD was recently discussed [32]. Primarily the 1:1 inclusion complexes were determined and the association constants were estimated with fluorescence method, conductivity measurements and ^19^F NMR measurements. The alkyl side chain on the imidazolium ring entered into the cavity of β-CD and the association constant was observed higher for the longer alkane chain [33]. For the chloride anion, it was shown that the anion’s inclusion in the cave is negligible [34]. Thus, the imidazolium ring does not enter the cavity of β-CD, but my interact with the β-CD outside surface.

### 2.2. IR Spectroscopy

The comparison of the transmittance intensity between β-CD infrared spectra (see Figure S1) and those of the pure IL and the mixture of (IL + β-CD) (see Figure S2) indicated that the formation of complexation with β-CD and the IL was confirmed by the lower transmittance of the mixture spectra.

**Scheme 1:**
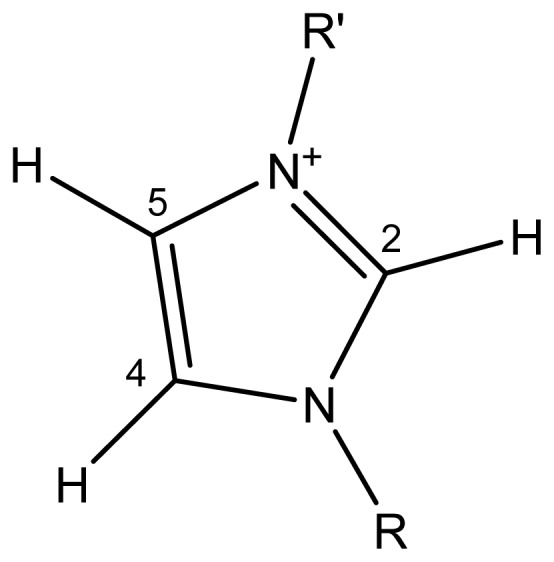
The structure of the imidazolium cation.

The aromatic C−H stretching vibrations in the imidazolium ring (see diagram below, Scheme 1) result in three highly characteristic infrared bands, between 3200 and 3050 cm^−1^, that depend on the presence and strength of hydrogen-bonding interactions between these protons and the anions of the IL [35].

The shift of these vibrations to higher wave numbers is caused by an increase of the C−H bonds strength in the imidazolium ring as their participation in hydrogen-bonding interactions increases. The shift of the stretching vibrations to higher frequencies in the imidazolium salts indicates that the hydrogen-bridging interactions are weakened by the substituent [36–38].

The sensitivity of the C-H frequencies to the strength of the hydrogen-bonding interaction between the IL and β-CD varies for different hydrogen atoms at the imidazolium ring. The *ν* [C(4)−H] and *ν* [C(5)−H] stay similar, but the *ν* [C(2)−H] change by more than 20–30 cm^−1^. This results in the relatively high sensitivity of *ν* [C(2)−H] to an increased participation of the imidazolium cation in the hydrogen bonding during the complexation. However, it is possible that β-CD also has OH vibrations that may overlap with H_2_O, and complicate the interpretation. In view of all the additions, the validity of IR as a tool for assessing the interactions of the IL with β-CD is thus a bit questionable. The conclusion can be made that the IR spectroscopy is not a very suitable tool to probe the complexation between the IL and β-CD in a presence even of a small amount of water.

### 2.3. Activity Coefficients at Infinite Dilution–Theoretical Basis

The equation developed by Everett [39] and Cruickshank *et al.* [40] was used in this work to calculate the $\gamma_{13}^{\infty }$ of solutes in the ionic liquid.

(1) $$\text{ln}\;\gamma_{13}^{\infty }=\text{ln}\left(\frac{n_{3}RT}{V_{\text{N}}P_{1}^{*}}\right)-\frac{P_{1}^{*}\left(B_{11}-V_{1}^{*}\right)}{RT}+\frac{P_{\text{o}}J_{2}^{3}\left(2B_{12}-V_{1}^{\infty }\right)}{RT}$$

The *V*_N_ denotes the net retention volume of the solute, *P*_o_ the outlet pressure, $P_{\text{o}}J_{2}^{3}$ the mean column pressure, *n*_3_ the number of moles of solvent on the column packing, *T* the column temperature, $P_{1}^{*}$ the saturated vapor pressure of the solute at temperature *T*, *B*_11_ the second virial coefficient of pure solute, $V_{1}^{*}$ the molar volume of the solute, $V_{1}^{\infty }$ the partial molar volume of the solute at infinite dilution in the solvent and *B*_12_ (where 2 refers to the carrier gas, helium), the mixed second virial coefficient of the solute and the carrier gas. The values of *B*_11_ and *B*_12_ were calculated using the McGlashan and Potter [41] equation for alkanes, and Tsonopolous [42] equation for the rest of solvents. Using the Hudson and McCoubrey combining rules [43,44], critical parameters for mixtures were calculated from the critical properties of the pure component.

The pressure correction term $J_{2}^{3}$ is given by

(2) $$J_{2}^{3}=\frac{2}{3}\frac{{\left(P_{\text{i}}/P_{\text{o}}\right)}^{3}-1}{{\left(P_{\text{i}}/P_{\text{o}}\right)}^{2}-1}$$

The net retention volume of the solute *V*_N_, is given by the equation

(3) $$V_{\text{N}}={\left(J_{2}^{3}\right)}^{-1}U_{\text{o}}\left(t_{\text{R}}-t_{\text{G}}\right)$$

where *t*_R_ and *t*_G_ are the retention times for the solute and an unreturned gas, respectively.

The vapor pressure values were calculated using equations and constants taken from the literature [45–47]. Critical data used to calculate *B*_11_ and *B*_12_ were obtained from literature [48,49].

### 2.4. Activity Coefficients at Infinite Dilution

Tables 1, 2, 3 and 4 list the $\gamma_{13}^{\infty }$ values for many solutes of different polarity as *n*-alkanes, cyclohexane, alk-1-ynes, aromatic hydrocarbons, alcohols, tetrahydrofurane (THF), tiophene, ethers, water, and many organic compounds in the four investigated ILs: [EMIM][Br], [BMPYR][Cl], [BMPYR][Br], and [OMIM][NTf_2_], in mixtures (IL + β-CD), or in pure β-CD in the temperature range from (338.15 to 398.15) K. Some solutes were measured only in the chosen IL, or in the mixture of (IL + β-CD) and sometimes only in one temperature because of the experimental problems. Some measurements for [EMIM][Br] and [BMPYR][Br] were performed for the subcold ILs. The substances as propionic acid, or butyric acid, or tetrahydronaphthalene, or chinolinum, and many others were used as a polar compounds with a longer retention times. The main object of these measurements was to analyze the interaction of the IL with different polar and non-polar solvents at infinite dilution, and the influence of the addition of small amount of the β-CD to the IL. These results were compared to those obtained for the pure β-CD. Aliphatic compounds show the highest values of $\gamma_{13}^{\infty }$, (*n*-alkanes, cycloalkanes, and alk-1-ynes). High values of $\gamma_{13}^{\infty }$ indicate very small interactions between solute and solvent. The values of $\gamma_{13}^{\infty }$ for series of solutes increase with an increase of the solute alkyl chain (see Tables 1, 2, 3 and 4). The interaction of triple bond and the hydrogen atom in alk-1-ynes with polar IL reveals lower values of $\gamma_{13}^{\infty }$ than those for *n*-alkanes with the same carbon number. Aromatic hydrocarbons have the smallest values of $\gamma_{13}^{\infty }$ from the rest of investigated hydrocarbons. This is caused by strong interactions between six *π*-delocalized electrons in benzene structure with the polar IL.

The high values of $\gamma_{13}^{\infty }$ were observed for *o*-, *m*- and *p*-xylene in [BMPYR][Br] (see Table 3). Aromatic compounds must interact, not only with the IL, but also with β-CD since the cavity of β-CD is hydrophobic. There is the competition in the solution between the IL and aromatic compound for the inclusion complexes with β-CD.

The lowest values of $\gamma_{13}^{\infty }$ are for alcohols and water. Slightly higher values are observed for thiophene, ketones and ethers, especially for tetrahydrofuran, THF. Alcohols additionally interact strongly via the hydroxyl group with the [BMPYR]^−^ and [EMIM]^−^ cation. Di-*n*-propyl ether even though contain oxygen in its structure do not interact as strongly as other polar compounds (high value of $\gamma_{13}^{\infty }$).

The highest interaction of solutes with the IL is observed for [OMIM][NTf_2_] (see Table 4). In pure β-CD the values of $\gamma_{13}^{\infty }$ for aliphatic hydrocarbons, cyclohexane, alkenes, and alk-1-ynes are higher than those in the pure IL [OMIM][NTf_2_] and mixtures of {[OMIM][NTf_2_] + β-CD} (see Table 4). This can be explained with the lower interaction of solutes with β-CD than with [OMIM][NTf_2_]. This is also evidence that octyl side chain offers better interaction with *n*-alkanes *via* the van der Walls interactions than β-CD does. Opposite, the values of $\gamma_{13}^{\infty }$ for aliphatic hydrocarbons e.g., *n*-decane in [EMIM][Br], [BMPYR][Cl], and [BMPYR][Br] are much higher (lower interaction) than those in pure β-CD. This is no doubt the result of the formation of stronger complexes of *n*-decane with β-CD than with examined ILs. These ILs are much more polar than [OMIM][NTf_2_] and they can offer lower interaction with aliphatic hydrocarbons. Different complexes may be found in the solution: the (IL + β-CD), the (IL + solute), or the (solute + β-CD). It depends on the polarity of the IL and the solvent. However, much lower values of $\gamma_{13}^{\infty }$ were found for the aromatic hydrocarbons, alcohols and thiophene in [EMIM][Br], [BMPYR][Cl], and [OMIM][NTf_2_] than those in β-CD. Structure of these compounds and spherical effects diminish the possibility of the complexation with β-CD. After all, it is well-known that ILs, in general, interact with aromatics extremely well, better than with β-CD.

Contrary to these effects, the addition of β-CD to the IL decreases the interaction with solutes in most of the ILs. For most of the solutes in [EMIM][Br], [BMPYR][Cl], [BMPYR][Br] and [OMIM][NTf_2_] the values of $\gamma_{13}^{\infty }$ were higher in the mixture of (IL + β-CD) than those in the pure IL (see Tables 1, 2, 3 and 4). For example for ethylbenzene $\gamma_{13}^{\infty }=13.3$ in β-CD; $\gamma_{13}^{\infty }=71.2$ in the mixture ([BMPYR][Cl] + β-CD), respectively at *T* = 338.15 K (see Table 2). This lower interaction of mixture (IL + β-CD) with solutes can be only explained by the strong inclusion effects between the IL and the β-CD itself. In general, we observed 30% higher values of $\gamma_{13}^{\infty }$ for the mixtures of (IL + β-CD) than that for pure IL. Only for water, the differences between the values of $\gamma_{13}^{\infty }$ in the IL and in the mixtures of (IL + β-CD) are not significant. It was observed, for two ILs, that the values of $\gamma_{13}^{\infty }$ of water were lower in the mixture of (IL + β-CD) than those in pure ILs [BMPYR][Br] and [OMIM][NTf_2_]. It has to be the specific interaction between three polar compounds and the competition between the IL and the solutes for the possible complexation by the β-CD. It can be expected that complexes of β-CD with the IL increases the interaction with water and similarly increases the solubility of these complexes in water. Thus, the IL may be used as co-solvent increasing the solubility of β-CD in water.

The influence of temperature on $\gamma_{13}^{\infty }$ values is shown in Figures S3 and S4. For [EMIM][Br], or the mixture of ([EMIM][Br] + β-CD) for example the values of the $\gamma_{13}^{\infty }$ of aromatic hydrocarbons, or alcohols mostly decreases with an increase of temperature. For [BMPYR][Cl], the values of the $\gamma_{13}^{\infty }$ of aliphatic and aromatic hydrocarbons decreases with an increase of temperature. Figure S3a,b show the natural logarithm of the activity coefficients in the ionic liquid, or in the mixture of (IL + β-CD) as a function of the inverse absolute temperature for the methanol in the chosen ionic liquid. Figure S4a,b present water in [BMPYR][Cl] and *o*-xylene in [OMIM][NTf_2_], respectively. The experimental points are close to the linear dependence in van’t Hoff coordinates, which is typically observed within short temperature intervals. Figures S3 and S4 show the influence of temperature on $\gamma_{13}^{\infty }$ and the influence of the addition of β-CD to the IL.

Table 5 [50–55] presents the selectivity parameter, calculated from the $\gamma_{13}^{\infty }$ values ( $S_{ij}^{\infty }=\gamma_{i}^{\infty }/\gamma_{j}^{\infty }$) for *n*-heptane/benzene, or cyclohexane/benzene, or *n*-decane/thiophene separation problems as well as the capacity of the IL (for benzene, or thiophene) ( $k_{j}^{\infty }=1/\gamma_{j}^{\infty }$). Presented data are for selected ILs, measured in this work, and from the literature at *T* = 338.15 K. The comparison was shown with sulfolane and NMP, which are used in industry [55]. From the selectivities and capacities presented in Table 5 it is easy to notice that the lowest values are presented for pure β-CD. The addition of β-CD to the IL increases the values of separation for presented mixtures in comparison with pure β-CD. However, the addition of β-CD to the IL decreases the separation parameter in comparison with the pure IL (see [OMIM][NTf_2_]). It confirms our interpretation of the competition of interaction between three compounds in the solution.

The ILs measured by us do not show very attractive $S_{ij}^{\infty }$ and $k_{j}^{\infty }$. For the *n*-heptane/benzene separation problem the best is the [OMIM][NTf_2_], for which $S_{ij}^{\infty }=10.6$. This is lower selectivity than those for [BMPYR][CF_3_SO_3_] ( $S_{ij}^{\infty }=33.6$) [50] or for [EMIM][SCN] ( $S_{ij}^{\infty }=96.0$) [51], and for sulfolane ( $S_{ij}^{\infty }=20.5$) [52]. Similar dependencies are observed for the cyclohexane/benzene separation problem with lower values of $S_{ij}^{\infty }$.

The only new and interesting result is observed for the *n*-decane/thiophene separation problem using [BMPYR][Cl], for which the value of $S_{ij}^{\infty }=158.9$ at *T* = 338.15 K. This is, however, a lower value than that for [BMIM][SCN] ( $S_{ij}^{\infty }=329.2$) [51]. For the [BMPYR][CF_3_SO_3_], the selectivity for the *n*-decane/thiophene separation problem was $S_{ij}^{\infty }=118.2$ [50].

One IL used by us was described earlier in the literature. The result of selectivity and of capacity for the *n*-heptane/benzene separation problem for [OMIM][NTf_2_] at lower temperature is very similar: $S_{ij}^{\infty }=9.9$ and $k_{j}^{\infty }=1.52$ at *T* = 323.15 K [56].

## 3. Experimental Section

### 3.1. Materials and Chemicals

β-Cyclodextrin (CAS 7585-39-9), >99 *w*/*w*% was obtained from Sigma-Aldrich and was dehydrated at 393 K under reduced pressure. The ILs have a purity of >0.98 mass fraction and were supplied by Solvionic, France. The ILs were further purified by subjecting the liquid to a very low pressure in a vacuum desiccator at temperature about 300 K for approximately 5 h. This procedure is supposed to remove any volatile chemicals and water from the ILs. The solutes used for the measurements of activity coefficients at infinite dilution were purchased from Sigma-Aldrich, 0.98+ and were used without the further purification because the GLC technique separated any impurities on the column. Water was bidistilled, deionized, and degassed.

### 3.2. Water Content

Water content was analyzed by using the Karl-Fisher titration technique (method TitroLine KF). The analysis showed that the water mass fraction in the ILs, was <850 × 10^−6^.

### 3.3. Solubility Measurements

As a first step, the qualitative results of the solubility of β-CD in ten ILs was tested. The mixture of 15 mol % of β-CD in the IL was heated during 10 min at temperature *T* = 423 K and the resulted phase equilibria was observed. The ILs used: [EMIM][Cl], [EMIM][Br], [BMIM][Cl], [EtOHMIM][Cl]; [BMPYR][Cl], [BMPYR][Br], [BMIM][BF_4_], [BMIM][PF_6_], [OMIM][NTf_2_], and [BMIM][CF_3_SO_3_].

The mixtures that revealed one-phase after the first treatment (the complete solubility) were chosen for the next step of the solubility measurements. The SLE temperatures were determined using the dynamic method described by us earlier [57].

The sample of known composition was placed in a glass thermostated vessel. The temperature of stirred vessel was slowly increased (less than 2 K h^−1^ near the equilibrium point) until the last crystals disappeared. The consecutive increasing and decreasing of temperature in the vicinity of the equilibrium value makes it possible to obtain the temperature corresponding to the saturation of the sample in equilibrium. The temperature was measured with an electronic thermometer. The accuracy of the temperature measurements was estimated to be ±0.1 K. The global uncertainty of β-CD solubility measurements was estimated to be ±0.0005 mole fraction in composition and ±1 K in temperature. The ILs used were: [EMIM][Cl], [EMIM][Br], and [BMIM][Cl]. The experimental results are presented in Figure 1a–c and in Tables S1 and S2.

### 3.4. IR Spectroscopy

IR spectroscopy was used to determine the interactions between β-CD and the IL in the binary solution. IR absorption spectra were obtained by using a Perkin Elmer Spectrum One with a Dynascan interferometer and an optical resolution of 0.5 nm. The quartz quvettes were used of volume 1 cm^3^. The samples were prepared by weighing the compounds in the concentration β-CD:IL = 0.15 in moles. Concentrations of the organic compounds were determined using the reference calibration curve.

### 3.5. Apparatus and Experimental Procedure

Inverse chromatography experiments were carried out using a Varian CP-3800 gas chromatograph equipped with a heated on-column injector and a flame ionization detector. The injector and detector temperatures were kept at *T* = 523 K during all experiments. The helium flow rate was adjusted to obtained adequate retention times. Exit gas flow rates were measured with an Alltech digital flow check mass flowmeter. The temperature of the oven was measured with a Pt 100 probe and controlled to within 0.1 K. A personal computer directly recorded detector signals, and corresponding chromatograms were obtained using Galaxie software (version 1.10; Agilent Technology: Santa Clara, CA, USA, 2011).

Column packing of 0.3 m to 1 m length containing β-CD, or the IL with β-CD, or the pure IL with different loading of stationary phases on Chromosorb W-AW (60–80 mesh) were prepared using the rotary evaporator technique. After evaporation of methanol (for the pure IL column) and/or the mixture of 85 mol% of methanol +15 mol% of ammonium water (to increase the solubility of cyclodextrin) for the other columns under vacuum, the support was equilibrated at *T* = 398 K over 6 h in the apparatus. The mass of the packing material was calculated from the mass of the packed and empty column and was checked during experiments. A small volume of samples of 1 to 5 μL were dozen to keep the system at infinite conditions. Each experiment was repeated at least twice to check the reproducibility. The data were obtained at three temperatures, *T* = 338.15 K, *T* = 368.15 K and *T* = 398.15 K. Retention times were generally reproducible to within 0.01–0.03 min. The measurements of retention times were repeated systematically every day for selected solutes to check the stability of the experimental conditions, such as the possible eluation of the stationary phase by the helium stream. No changes in the retention times were observed during two months of continuous operation.

## 4. Conclusions

The interaction of β-CD with the IL was presented by the measurements of the SLE, IR spectroscopy, and activity coefficients at infinite dilution. It was shown that the investigated in this work ILs could form inclusion complexes with β-CD. The interaction between the IL and β-CD was confirmed in the SLE diagrams in form of eutectic points. The measurements of the activity coefficients at infinite dilution for many solutes in pure ILs, mixtures of (IL + β-CD) and in pure β-CD revealed different interaction between solutes and solvents. The investigated in this work ILs present low selectivities and capacities in *n*-heptane/benzene, cyclohexane/benzene, or *n*-decane/benzene separation problems in comparison with those observed in other popular ILs. The mixtures of (ILs + β-CD) did not improve selectivity for the chosen separation problem. The only interesting selectivity was found for [BMPYR][Cl] for the separation of *n*-decane and thiophene. The addition of the β-CD to the IL changes the polarity and basicity of the mixture in comparison with the pure IL. It changes the solubility of organic compounds in the mixture. Complexes formed by the IL with β-CD decreases the interaction of solutes with IL. This minimizes the possible interaction of β-CD with solutes.

The measurements of activity coefficients confirmed that in the ternary system (IL + β-CD + water) different mechanism produces inclusion complexes. The solubility of β-CD in water may be increased by use of co-solvent–ionic liquid.

## Supplementary Information



## Figures and Tables

**Table 1:** The experimental activity coefficients at infinite dilution, $\gamma_{13}^{\infty }$ for the solutes in the IL [EMIM][Br] (12.20 *w*%) and IL {[EMIM][Br] (12.20 *w*%) + β-CD (17.42 *w*%)} at different temperatures.

Solute	*T*/K					

	338.15	338.15 a	368.15	368.15 a	398.15	398.15 a
*n*-decane	93.7					
hept-1-yne	14.7					
oct-1-yne	23.9	26.4				
benzene	2.61	1.79		2.16		
toluene	5.21	7.39	5.15	8.31		
ethylbenzene	10.8	17.5	8.15	15.4		
*o*-xylene	9.07	16.3	8.23	14.5		
*m*-xylene	12,0	13.6	9.28	18.4		
*p*-xylene	10.3	18.4	10.7	14.5		
methanol	0.212	0.378	0.167	0.304	0.229	0.232
ethanol	0.284	0.965	0.289	0.888	0.613	0.532
propan-1-ol	0.457	1.42	0.429	1.2	0.954	0.92
butan-1-ol	1.03	1.99	0.767	1.66	1.75	1.28
THF	3.01	3.07				
tiophene	1.34	1.73	1.35	0.637		
acetone	1.18	3.43				
pentan-2-one	5.22	7.93	4.53			
water		0.336	0.096	0.416	0.257	0.196
propionic acid				0.244	0.209	0.193
tetrahydronaphthalene				41.1		34.8
butylamine					0.796	0.835
butyric acid					0.33	0.346
chinolinium					6.36	2.8
pyridinium					2.77	1.94

a Column for mixture of ([EMIM][Br] + β-CD).

**Table 2:** The experimental activity coefficients at infinite dilution, $\gamma_{13}^{\infty }$ for the solutes in the IL [BMPYR][Cl] (15.75 *w*%) and IL {[BMPYR][Cl] (15.76 *w*%) + β-CD (14.20 *w*%)} at different temperatures.

Solute	*T*/K					

	338.15	338.15 a	368.15	368.15 a	398.15	398.15 a
*n*-decane	259					
hept-1-yne	13.4					
oct-1-yne	18.2		13.2			
benzene	4.08		3.11			
toluene	7.51		5.30			
ethylbenzene	13.3	71.2	8.99			
*o*-xylene	8.90	73.9	7.86	30.9		
*m*-xylene	13.6		9.78			
*p*-xylene	11.9		9.19			
methanol	0.098	0.619	0.116	0.590	0.112	0.594
ethanol	0.200	1.44	0.228	1.22	0.191	1.18
propan-1-ol	0.254	2.18	0.260	1.71	0.248	1.68
butan-1-ol	0.328	2.66	0.331	2.33	0.343	2.43
THF	4.47					
tiophene	1.63	5.19	1.55	3.12		
acetone	2.72					
pentan-2-one	6.37		5.05			
water	0.107	0.354	0.089	0.429	0.086	0.455
propionic acid				1.32	0.210	1.20
tetrahydronaphthalene			15.2	30.1	14.4	28.2
butylamine					0.380	0.691
butyric acid						0.381
chinolinium					1.26	3.54
pyridinium					0.994	1.90

a Column for mixture of ([BMPYR][Cl] + β-CD).

**Table 3:** The Experimental activity coefficients at infinite dilution, $\gamma_{13}^{\infty }$ for the solutes in the IL [BMPYR][Br] (15.73 *w*%) and IL {[BMPYR][Br] (14.95 *w*%) + β-CD (14.81 *w*%)} at different temperatures.

Solute	*T*/K					

	338.15	338.15 a	368.15	368.15 a	398.15	398.15 a
*n*-nonane		167				
*n*-decane	115	213				
ethylbenzene		35.5				
*o*-xylene		49.0		26.3		
*m*-xylene		50.0				
*p*-xylene		48.2				
methanol		0.68		0.594		0.548
ethanol		2.01		1.58		1.25
propan-1-ol		3.41		2.43		1.70
butan-1-ol		4.48		3.37		2.44
tiophene		7.1				
water	0.836	0.396	0.899	0.479	1.52	0.085
propionic acid			0.515	1.27	0.632	0.412
tetrahydronaphthalene				29.4		21.2
butylamine						0.648
butyric acid					0.685	0.663
chinolinium					7.97	2.39
pyridinium					0.18	2.25

a Column for mixture of ([BMPYR][Br] + β-CD).

**Table 4:** The experimental activity coefficients at infinite dilution, $\gamma_{13}^{\infty }$ for the solutes in the IL [OMIM][NTf_2_] (19.82 *w*%), IL {[OMIM][NTf_2_] (19.82 *w*%) + β-CD (9.04 *w*%)} and pure β-CD at different temperatures.

Solute	*T*/K						

	338.15	338.15 a	338.15 b	368.15	368.15 a	398.15	398.15 a
*n*-hexane							
*n*-heptane	4.28	9.16	9.44				
*n*-octane	4.41	12.0	13.6	5.37	9.99		
*n*-nonane	5.99	14.4	20.6	4.94	13.0		
*n*-decane	2.36	17.9	29.6	7.27	16.4		
cyclohexane	2.06	5.06	8.22				
hex-1-ene	1.91	5.14					
hept-1-yne	1.22	3.44	7.26	1.41	3.29		
oct-1-yne	1.54	4.22	9.61	1.96	4.21		
benzene	0.404	1.15	6.58	0.375	1.17		
toluene	0.539	1.51	9.34	1.10	1.58		
ethylbenzene	0.750	2.02	16.2	0.742	2.12		
*o*-xylene	0.506	1.79	21.5	0.544	1.94	0.572	1.89
*m*-xylene	0.685	1.91	17.4	0.724	2.13		
*p*-xylene	0.693	1.94	16.5	0.709	2.18		
methanol	0.384	1.21	0.703	0.288	1.10	0.161	0.724
ethanol	0.733	1.78	4.36	0.721	1.31		
propan-1-ol	0.803	1.94	6.98	0.514	1.76		
butan-1-ol	0.808	2.20	5.9	0.669	1.85		
THF	0.300	0.819	2.52	0.244	0.841		
dipropyl ether	1.38	3.89	3.77				
tiophene	0.364	1.09	4.03	0.323	1.11		
acetone	0.215	0.642		0.192	0.654		
pentan-2-one	0.288	0.858	5.02	0.293	0.932		
water	1.21	1.04		0.860	0.551		
propionic acid			70.1	0.433	1.17	0.290	0.687
tetrahydronaphthalene						8.7	2.75
butylamine			4.38			0.229	0.344
butyric acid						4.63	1.40
chinolinium						0.217	0.936
pyridinium						0.265	0.750

a Column for mixture of ([OMIM][NTf_2_] + β-CD);

b Pure β-CD.

**Table 5:** Selectivities, $S_{ij}^{\infty }$ and capacities, $k_{j}^{\infty }$ at infinite dilution for different ionic liquids and the different separation problems at *T* = 338.15 K.

Solvent	$S_{ij}^{\infty }=\gamma_{i}^{\infty }/\gamma_{j}^{\infty }$		$k_{j}^{\infty }=1/\gamma_{j}^{\infty }$	$S_{ij}^{\infty }=\gamma_{i}^{\infty }/\gamma_{j}^{\infty }$	$k_{j}^{\infty }=1/\gamma_{j}^{\infty }$

	*n*-heptane/benzene	cyclohexane/benzene	benzene	*n*-decane/thiophene	thiophene
[EMIM][Br]				69.9	0.75
[BMPYR][Cl]				158.9	0.61
[BMPYR][Br] + β-CD				30.0	0.14
[OMIM][NTf_2_]	10.6	5.1	2.48	21.3	2.75
[OMIM][NTf_2_] + β-CD	8.0	4.4	0.87	16.4	0.92
β-CD	1.4		0.15	7.3	0.25
[BMPYR][CF_3_SO_3_] a	33.6	12.1	0.68	118.2	0.91
[EMIM][SCN] b	96.0	22.3	0.28		
[BMIM][SCN] c	76.6	16.7	0.45	329.2	0.77
[1,3-BMPY][CF_3_SO_3_] d	22.8	8.7	0.79	85.6	1.07
Sulfolane e	20.5 f	6.9 f	0.43 f		
NMP g		5.9 f	0.94 f		
NMP + 6% (w/w) water g		7.3 f	0.50 f		

a From Reference [50];

b From Reference [51];

c From Reference [52];

d From Reference [53];

e From Reference [54];

f At temperature *T* = 333 K;

g From Reference [55].
