# Supplementary material for: Physico-Chemical Properties and Phase Behavior of the Ionic Liquid-β-Cyclodextrin Complexes
Source: Int J Mol Sci. 2013 Aug 13;14(8):16638–55. doi: 10.3390/ijms140816638 (PMC3759929; doi:10.3390/ijms140816638)
Supplement: Supplementary file 1 [file ijms-14-16638-s001.pdf]

Supplementary Information

Table S1. Experimental Solid-Liquid Phase Equilibria of Binary Systems {IL (1) + β-CD (2)}.

x_1	T/K	x_1	T/K
		[EMIM][Cl]	
0.8065	403.15	0.9709	326.15
0.8547	373.15	0.9804	337.15
0.9009	328.15	0.9901	348.15
0.9524	298.15	1.0000	358.15
0.9615	308.15		
		[EMIM][Br]	
0.8547	321.15	09709	305.15
0.9009	313.15	0.9804	324.15
0.9524	297.15	0.9901	341.15
0.9615	293.15	1.0000	350.15
		[BMIM][Cl]	
0.8065	423.15	0.9615	338.15
0.8547	413.15	0.9709	318.15
0.9009	373.15	0.9804	303.15
0.9242	365.15	0.9901	321.15
0.9524	348.15	1.0000	338.15

Table S2. Experimental Eutectic Points of Binary Systems {IL (1) + β-CD (2)}.

IL	$x_{1,e}$	$T_{1,e}$/K
[EMIM][Cl]	0.940	275.0
[EMIM][Br]	0.961	293.1
[BMIM][Cl]	0.978	302.5

The IR Spectroscopy

Table S3. Infrared spectroscopic data for the aromatic stretching region (3200–3000 cm^{-1}), corresponding with C-H stretching frequencies in the imidazolium cation.

IL	ν[C(5)−H]	ν[C(4)−H]	ν[C(2)−H]
[EMIM][Cl]	3149 m	3092 m	3060 l
[EMIM][Cl] + β-CD	3150 m	3095 m	3085 m
[EMIM][Br]	3146 m	3072 m	3018 l
[EMIM][Br] + β-CD	3148 m	3088 m	3042 s
[BMIM][Cl]	3141 l	3064 m	3010 l
[BMIM][Cl] + β-CD	3146 l	3086 m	3049 s

Figure S1. IR spectra of β-CD.

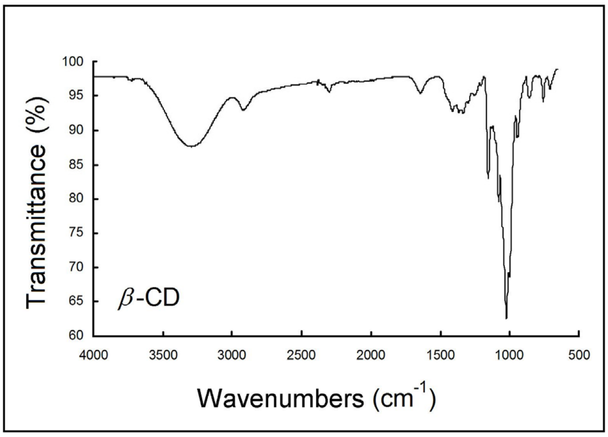

Figure S2. IR spectra of pure ILs and their mixtures with β-CD: (**a**) [EMIM][Cl]; (**b**) [EMIM][Cl] + β-CD; (**c**) [EMIM][Br]; (**d**) [EMIM][Br] + β-CD; (**e**) [BMIM][CL]; (**f**) [BMIM][CL] + β-CD; (**g**) [BMPyr][Cl]; (**h**) [BMPyr][Cl] + β-CD.

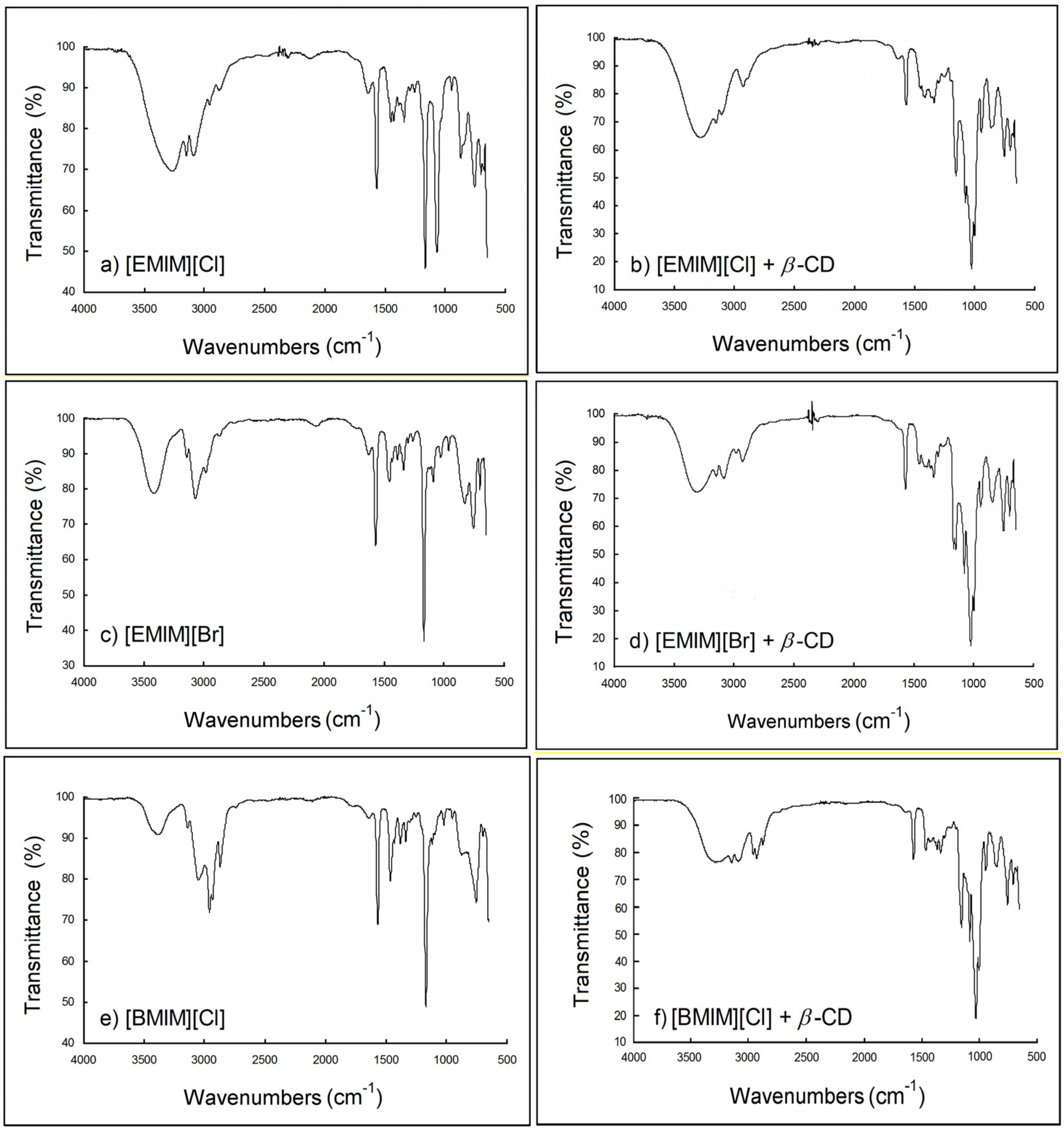

Figure S2. *Cont.*

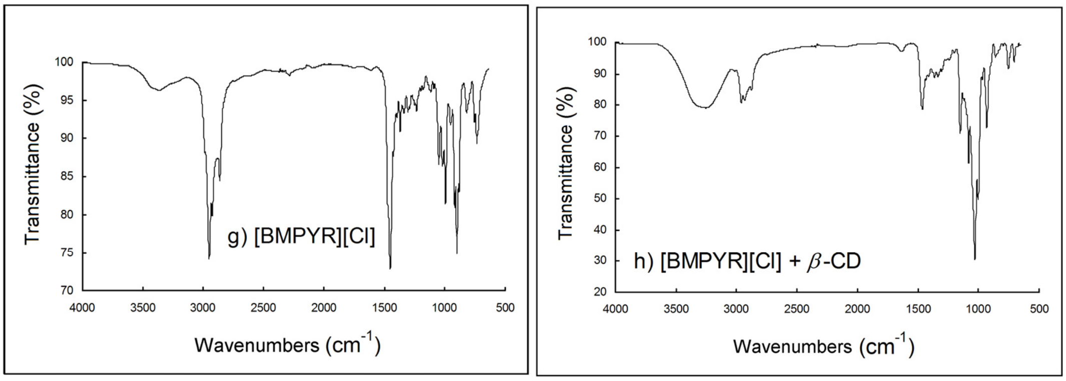

Figure S3. Plot of ln(γ_{13}^{∞}) *versus* $1/T$ for methanol in different ionic liquids and their mixtures with β-CD: (**a**) [BMPYR][Cl]; (**b**) [OMIM][NTf_2].

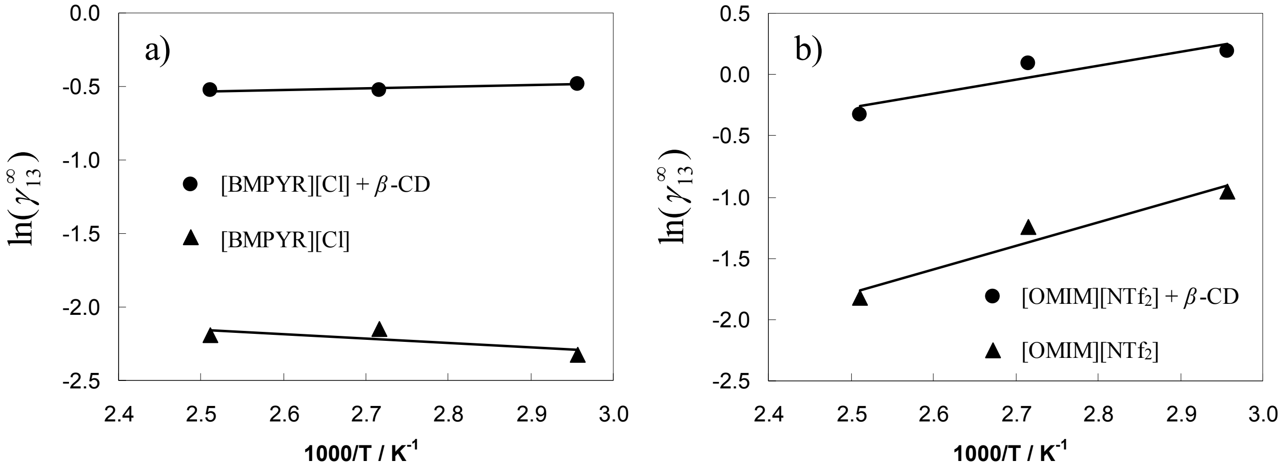

Figure S4. Plot of ln(γ_{13}^{∞}) *versus* $1/T$ for solutes in different ionic liquids and their mixtures with β-CD: (**a**) water + [BMPYR][Cl]; (**b**) *o*-xylene + [OMIM][NTf_2].

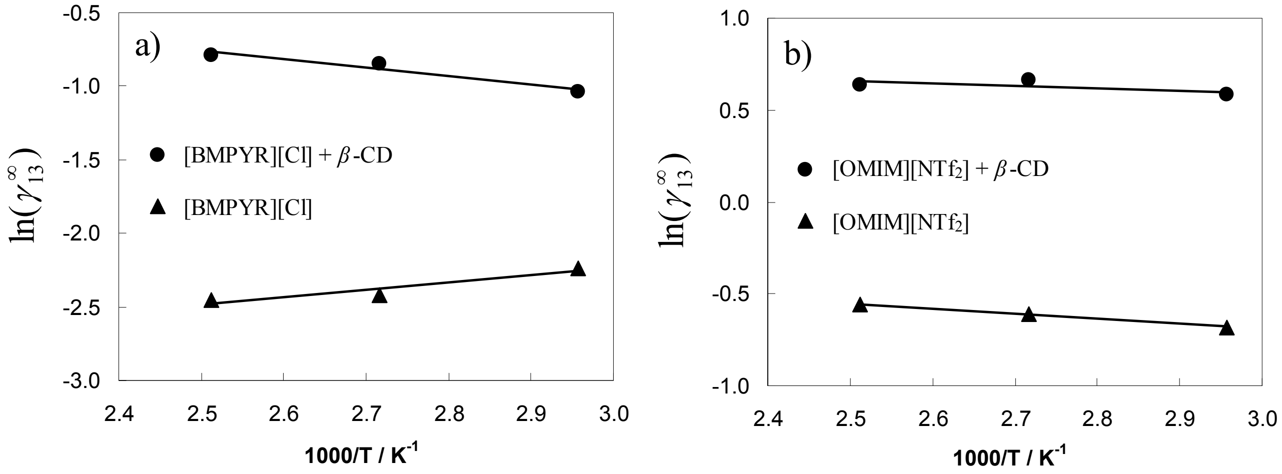

© 2013 by the authors; licensee MDPI, Basel, Switzerland. This article is an open access article distributed under the terms and conditions of the Creative Commons Attribution license (http://creativecommons.org/licenses/by/3.0/).
